# Enhance triangular fuzzy parametric framework for solid multi objective transportation problem with split decision variables

**DOI:** 10.1038/s41598-025-10949-4

**Published:** 2025-07-28

**Authors:** Vishwas Deep Joshi, Medha Sharma, Lenka Čepová, Huda Alsaud, Kanak Kalita

**Affiliations:** 1 Department of Mathematics, Faculty of Science, JECRC University, Jaipur, Rajasthan India; 2 Department of Machining, Assembly and Engineering Metrology, Faculty of Mechanical Engineering, VSB-Technical University of Ostrava, 70800 Ostrava, Czech Republic; 3 Department of Mathematics, College of Science, King Saud University, P. O. Box-22452, 11495 Riyadh, Saudi Arabia; 4 Department of Mechanical Engineering, Vel Tech Rangarajan Dr. Sagunthala R&D Institute of Science and Technology, Avadi, 600062 India

**Keywords:** Fuzzy programming, Exponential membership function, Preferred compromise solution, Fuzzy parameter based multi objective transportation problem, Accuracy parameter, Euclidean distance, Engineering, Mathematics and computing

## Abstract

This paper introduces a novel two-step generalized parametric approach for addressing Fuzzy Multi-Objective Transportation Problems (FMOTPs), commonly encountered in logistics and transportation systems when essential parameters—such as supply, demand, and transportation costs—are uncertain. Driven by the necessity for resilient and flexible decision-making amidst uncertainty, the method employs Triangular Fuzzy Numbers (TFNs) and an accuracy parameter μ ∈ [0,1] to turn fuzzy data into precise equivalents through parametric transformation. Initially, imprecise input data are methodically converted into a sequence of Crisp Multi-Objective Transportation Problems (CMOTPs). In the subsequent phase, these CMOTPs are addressed by Fuzzy Linear Programming (FLP), and the most equitable solution at each μ-level is determined by its Euclidean distance from the fuzzy ideal solution. The suggested method is tested by numerical case studies and compared with current models—such as Nomani’s approach, fuzzy DEA, and Grey Relational Analysis (GRA)—showing enhanced performance in optimality proximity, solution stability, and ranking accuracy. This research has practical applications, including improved managerial capacity to manage uncertainty, reconcile trade-offs among cost, time, and service quality, and execute robust transportation strategies in fluctuating environments. The model’s scalability and openness make it suited for integration into enterprise logistics systems across industries such as manufacturing, retail, distribution, and e-commerce. The study offers a systematic and computationally efficient framework that enhances both theoretical comprehension and practical implementation of fuzzy optimization in multi-objective transportation planning.

## Introduction

Linear programming problems play a crucial role in transportation^1^ and are widely applied in various domains, including inventory management, supply chain optimization, logistics systems, and production planning. In a conventional transportation problem, cost, supply, and demand are the key determining factors. However, in today’s highly competitive market, these criteria may not always be well-defined. The price of a product may vary frequently or depend on the manufacturing method. Moreover, the lack of pertinent data regarding product shipments can lead to an ambiguous and indistinct correlation between supply and demand. Zadeh^2^ introduced the notion of fuzziness to tackle these circumstances and simplify the management of unclear data. In many fields, such as economics, psychology, philosophy, mathematics, and statistics, the decision-making process is essential. Transportation is essential in distribution networks, facilitating the efficient transfer of goods. The transportation problem’s (TP) main objective is to reduce delivery expenses while allowing producers to efficiently satisfy consumer demand. The transportation problem (TP) encompasses the factors of cost, availability, and market requirements. Commercial commodities can be transported between different locations utilizing various techniques within a transportation network, therefore enabling cost savings and adherence to time constraints. Initially presented by Hitchcock^3^, the first fundamental theoretical principles (TPs) were subsequently subjected to thorough examination by several authors, as evidenced in the literature.

The transportation infrastructure is vital for promoting the economic growth of a country. The transportation sector is crucial for the advancement of a nation as it facilitates the transportation of commodities, individuals, machinery, equipment, and life-saving provisions to their designated destinations. Examples of this phenomenon encompass the conveyance of merchandise or individuals, the delivery of machinery equipment to manufacturing facilities, and the dissemination of sustenance and vital provisions to locations experiencing deprivation.

The transportation problem can be considered a specialized case of the Linear Programming Problem (LPP) framework. The goal is to optimize multiple objectives by determining the quantity of products to be transported from various sources to different destinations while adhering to specific constraints.

**Fig. 1:**
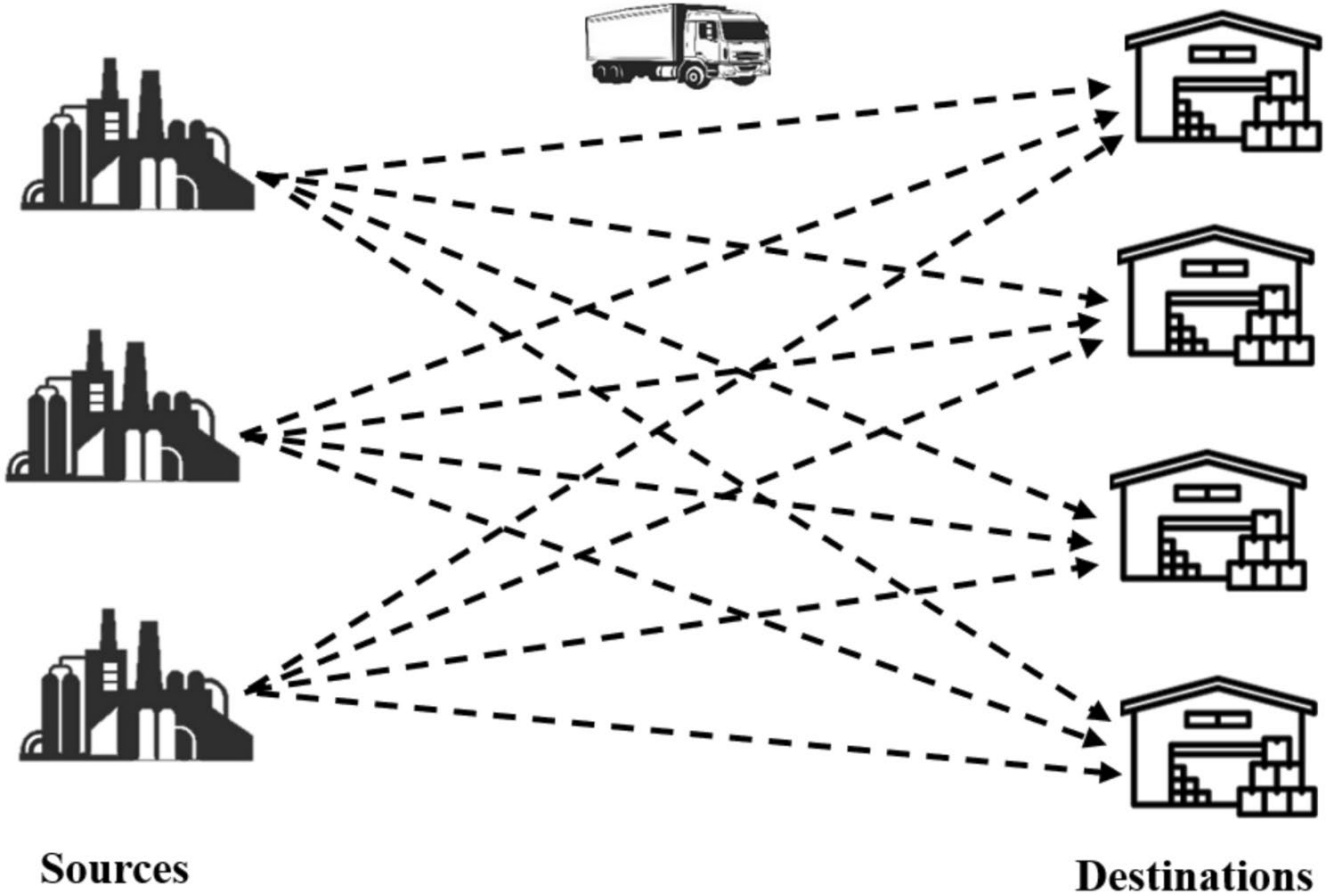
Depiction of the fuzzy transportation problem.

The fuzzy transport problem (FTP) is a variant of the traditional transportation problem in which the values of supply, demand, and expenses are expressed as fuzzy numbers rather than precise values (Fig. 1). This approach is employed in situations where there is ambiguity or lack of accuracy in the data. The fuzzy multi-objective transportation problem (FMOTP) is a variant of the fuzzy transportation problem that includes the consideration of several competing objectives. In this problem, characteristics such as supply, demand, costs, and objectives are expressed as fuzzy numbers.

Typically, conventional transportation problems are addressed with the assumption that all relevant characteristics are precisely known. However, when any situation that exists in real-world situations is simulated two primary challenges arise. The first issue is the presence of contradictory goals in the problem definition, while the secondary concern is the ambiguity in the description of the problem parameters. The resolution of the model’s mathematical expression using conventional techniques is hampered by the imprecision in the parameters. As a result, there has been a rise in imprecise parameters and the use of different fuzzy approaches to deal with these uncertainties. In 1970, Bellman and Zadeh initially proposed the concept of fuzzy set theory within the framework of challenges encountered in decision-making. Subsequently, a multitude of academics have showed considerable interest in this specific field and developed a diverse array of strategies and methodologies employing fuzzy concepts to efficiently tackle transportation challenges that are marked by uncertainty. The application of fuzzy linear programming methods was suggested by Zimmermann^4^ as a viable strategy for addressing the FTP. These principles have developed into several fuzzy optimization methods that offer a structure for solving finite term problems (FTPs) with one or more objectives^5^.

The Fuzzy DEA method simplifies the analysis of MOTPs with uncertain costs by transforming them into single objective problems^6^. Fuzzy approach outperforms traditional methods in handling the "more-for-less" paradox in multi-objective transportation problems. A multi-choice fractional transportation problem with multiple objectives was solved using Newton’s interpolation method to address uncertainty^7,8^. The multi-objective, uncertain transportation problem with random parameters is addressed by Newton’s interpolation^7,8^. Fuzzy and goal programming approach optimizes uncertain multi-objective transportation decisions in manufacturing^9^. Uncertainty theory addresses limitations of probability and fuzzy sets in modelling human-expressed uncertainty^10^. Sakawa^11^ developed interactive computer programs to address fuzzy multi-objective linear programming challenges. The study conducted by Carlsson and Korhonen^12^ investigated the substantial influence of FLP and parametric programming on the resolution of linear programming problems (LPP) characterized by precise parameter values. The Fuzzy harmonic mean approach is proposed by Kacher and Singh^13^ as an excellent approach for addressing completely Friction Mode Optimization problems. Mardanya and Roy^14^ addressed the fuzzy multi-objective multi-item solid transportation problem using interval and rough interval methods. These techniques effectively account for uncertainty and variability, providing a more robust framework for analysing transportation decisions. Nearest interval approximation solves fully MOTP^15^. Efficient optimization of uncertain multi-objective transportation problems is achieved via stochastic and compromise programming^7,8^. Weighted goal programming is a technique that Nomani et al.^16^ suggest using to solve multi-objective transportation problems (MOTPs).

The book introduces a unified framework for optimizing complex engineering systems under uncertainty using multidisciplinary design techniques, combining probabilistic models, surrogate methods, and reliability-based strategies^17^. This paper proposes a reliability evaluation and optimization method for precision hot extrusion automatic production lines, utilizing fault tree analysis and fuzzy analysis theory to identify and improve critical links affecting efficiency and quality^18^. This paper presents a novel solution concept for interval-valued matrix games by transforming them into multi-objective problems and addressing them through an intuitionistic fuzzy optimization technique featuring nonlinear membership and non-membership functions, thereby exhibiting enhanced efficacy compared to existing methods^19^. This paper presents an Intuitionistic Fuzzy Optimization (IFO) method for addressing matrix games under uncertainty by establishing intuitionistic fuzzy (IF) objectives for payoffs, which encompass levels of satisfaction and rejection, subsequently transforming the issue into two manageable linear programming problems^20^. This study proposes a fuzzy risk-based hybrid MCDM approach integrating BWM, SIR, and TOPSIS to optimize multimodal transportation decisions, effectively addressing complexity, uncertainty, and risk in route selection^21^. This study introduces a Triple Vague framework using T-spherical fuzzy sets and the SIR-MCDM approach to effectively select the optimal industry for future development in the context of evolving industrial revolutions^22^. Table 1 presents a comparative analysis of research trend for solving MOTP under uncertainty.

**Table 1:** Comparative analysis of research trend for solving MOTP under uncertainty.

Authors	Year	Dimension	Objective	Environment	TC	TP	VOS	Solution method
Zimmermann^4^	1978	1	2			Yes		FLP technique
Bagheri et al.^23,24^	2020	2	3	Fuzzy	Yes	Yes	Yes	DEA approach
Kacher and Singh^13^	2022	2	3	Fuzzy	Yes	Yes		Fuzzy harmonic mean technique
Niksirat^15^	2022	2	2	LR fuzzy	Yes			Nearest interval approximation
Mardanya and Roy^14^	2023	3	2	Trapezoidal fuzzy number	Yes			Interval programming, fuzzy programming and expected value operator
Joshi et al.^9^	2023	2	3	uncertain	Yes			Fuzzy approach
Kacher and Singh	2023	2	3	Fuzzy	Yes	Yes	Yes	GRA
Joshi et al.^7^	2024	3	2	Multi-choice	Yes	Yes		NDD
Joshi et al.^8^	2024	3	2	Multi-choice	Yes			NDD
This article	2024	2	3	Fuzzy	Yes	Yes	Yes	FLP technique

The fuzzy transportation problem is an expansion of the traditional transportation problem that integrates fuzzy logic notions to tackle uncertainties and imprecisions in real-world scenarios. Its importance stems from several factors

- In real-life scenarios, transportation costs, supply, and demand are often not precisely known. Variations in these values can be attributed to a range of factors including fuel prices, weather conditions, and market dynamics. Fuzzy transportation problems provide a more realistic representation of these uncertainties.
- By using fuzzy sets to represent costs and constraints, decision-makers can incorporate their subjective judgments and preferences into the model. This flexibility allows for more nuanced and adaptable solutions.
- In many situations, exact data may not be available or may be too costly to obtain. Fuzzy transportation problems can work with approximate or linguistic data, making them useful when precise information is lacking.
- By considering the fuzziness of parameters, these models facilitate better risk assessment and management in transportation and logistics planning.
- Fuzzy transportation problems can lead to more robust and reliable solutions compared to classical models, especially in dynamic and uncertain environments.
- The concept of fuzzy transportation problems extends beyond just logistics. This technology finds utility in diverse domains, including supply chain management, production planning, and resource allocation.
- Fuzzy models can be more easily adjusted to accommodate changes in the business environment, making them valuable for long-term planning and strategy.
- Fuzzy transportation problems can be combined with other optimization techniques, such as genetic algorithms or neural networks, to create hybrid models that leverage the strengths of multiple approaches.
- By providing more flexible and realistic solutions, fuzzy transportation problems can lead to more cost-effective decisions in the long run, potentially saving organizations significant resources.

This article proposes a strategy for addressing the fuzzy multi-objective transportation issue using two sequential procedures. To find the entire range of values connected to the fuzzy parameters, parametric programming is initially used. This method converts the fuzzy model into distinct crisp models, each aligned with a particular accuracy parameter value within the interval of 0 to 1. The second phase involves the use of the fuzzy linear programming approach to concurrently identify the compromise optimal solutions for the provided problem. Despite the multitude of solutions presented for addressing transportation problems under uncertainty, several significant shortcomings persist in the literature.

- Conventional linear programming and early fuzzy models frequently presume simplified or symmetric uncertainty frameworks. Nonetheless, actual logistics systems often display intricate, asymmetric, and non-linear uncertainties that traditional models are unable to encompass.
- Although many studies have investigated fuzzy multi-objective transportation problems (FMOTPs), numerous approaches address goals individually or depend on single-objective transformations, thereby constraining solution diversity and undermining quality.
- Despite the considerable potential of parametric programming to address fuzziness via the incremental adjustment of membership levels, its comprehensive application in FMOTP is still largely unexamined, especially concerning its integration with fuzzy linear programming for the selection of compromise solutions.
- Numerous current models exhibit a deficiency in thorough benchmarking against other recognized methodologies (e.g., fuzzy DEA, GRA, or weighted goal programming) across various levels of uncertainty.

This study tackles these deficiencies by establishing a robust, parametric, and multi-objective framework specifically designed to replicate real-world fuzziness and deliver meaningful, data-driven conclusions.

This study is motivated by the increasing need for decision-support systems in transportation planning that can function effectively amidst significant ambiguity and conflicting agendas. As global logistics evolve to be more integrated and dynamic, decision-makers must address

- Transportation expenses are subject to fluctuations due to fuel prices, geopolitical factors, and seasonal demand.
- Insufficient or inaccurate supply and demand data.
- The necessity to concurrently optimize various objectives (e.g., cost, time, and service level) instead of concentrating on a singular performance indicator.

Current models frequently do not provide solutions that encapsulate this complexity. Consequently, a novel methodology is required—one that is not only mathematically robust but also flexible, computationally efficient, and adept at encapsulating the inherent uncertainty in transportation networks. This research responds to that requirement by merging parametric analysis with fuzzy optimization, creating a more complete framework for current decision-making in logistics.

This study presents the subsequent key contributions:

1. The work presents an innovative two-step process in which fuzzy parameters are initially converted into precise models utilizing accuracy parameters μ ∈ [0, 1], subsequently employing fuzzy linear programming to determine compromise best solutions at different levels of confidence.
2. The model utilizes exponential membership functions to capture nonlinear sensitivity and more accurately represent decision-maker preferences in uncertain contexts than linear or symmetric alternatives.
3. The study does a comprehensive sensitivity analysis across several μ values and split-level scenarios, providing insights into the behavior and stability of solutions under different levels of fuzziness.
4. The suggested method is empirically evaluated against models including Nomani’s weighted goal programming, fuzzy DEA^23,24^, and GRA, exhibiting higher efficacy in producing solutions that approximate the ideal more closely.
5. The methodology is evaluated using numerical examples based on real-world fuzzy transportation problems, underscoring its practical significance and versatility.
6. Employing Euclidean distance to evaluate fuzzy compromise solutions introduces a logical and comprehensible aspect to decision assistance, assisting practitioners in selecting solutions that are both optimal and feasible.

The present study paper is organized in the following manner: Sect. 2 presents the underlying assumptions and notational representations. Section 3 provides fundamental definitions. In Sect. 4, the mathematical formulation of the FMOTP is presented. Section 5 introduces the fuzzy linear programming (FLP) approach. The approach put forward in Sect. 6. Section 7 includes a numerical example that showcases the practical implementation of the proposed methods. Analysis and conclusions are presented in Sect. 8. Section 9 presents the final conclusion.

## Basic definitions

Table 2 presents the abbreviations and notation used in this paper.

**Table 2:** List of abbreviations and notations.

Abbreviations	Full form
FLP	Fuzzy linear programming
FPP	Fuzzy parametric programming
GRA	Grey relationship analysis
FMOTP	Fuzzy multi-objective transportation problem
DEA	Data envelopment analysis
LPP	Linear programming problem
TP	Transportation problem
CMOTP	Crisp multi-objective transportation problem
FTP	Fuzzy transportation problem
LS	Left split
RS	Right split
TFN	Triangular fuzzy number
FCS	Fuzzy compromise solution
FIS	Fuzzy ideal solution
TOPSIS	Technique for order of preference by similarity to ideal solution
$R$	Number of objective functions
$m$	Enumeration of the sources of the transportation problem
$n$	Number of destinations of the transportation problem
${a}_{i}$	Supply at the ${i}^{th}$ origin
${b}_{j}$	Demand at the ${j}^{th}$ destination
${\chi }_{ij}$	The volume carried from the ${i}^{th}$ origin to ${j}^{th}$ destination
${U}_{r}$	Upper bound of the ${r}^{th}$ objective function
${L}_{r}$	Lower bound of the ${r}^{th}$ objective function
${Z}_{r}$	${r}^{th}$ objective function
$\mu$	Accuracy parameter

### Definition 3.1

Fuzzy set $\widetilde{A}$ real number that meets the criteria of normalcy and convexity is referred to as a fuzzy number^24^**.**

### Definition 3.2

Normality of a fuzzy set A is defined as the presence of at least one point $\chi \in X$ such that ${\mu }_{A}\left(\chi \right)=1$^24^**.**

### Definition 3.3

A fuzzy set A in ${\mathbb{R}}$ (real line) is an ordered pair collection $A=\left\{\left(\chi ,{\mu }_{A}\left(\chi \right)\right)|\chi \in {\mathbb{R}}\right\},$ where ${\mu }_{A}\left(\chi \right)$ is the fuzzy set membership function^24^**.**

### Definition 3.4^24^

If relation (1) provides the membership function of a fuzzy number $\widetilde{A}$, represented as $\widetilde{A}=({a}_{l},{a}_{m},{a}_{u})$, then $\widetilde{A}$ is referred to as a triangular fuzzy number.

1 $${\mu }_{\widetilde{A}}\left(\chi \right)=\left\{\begin{array}{c}\frac{\chi -{a}_{l}}{{a}_{m}-{a}_{l}} for {a}_{l}\le \chi \le {a}_{m}\\ \frac{{a}_{u}-\chi }{{a}_{u}-{a}_{m}} for {a}_{m}\le \chi \le {a}_{u}\end{array}\right.$$

### Definition 3.5^24^

An $\widetilde{A}=({a}_{l},{a}_{m},{a}_{u})$ triangular fuzzy number is considered non-negative (positive) if and only if ${a}_{l}\ge 0$.

### Definition 3.6^24^

The equality of two triangular fuzzy numbers $\widetilde{A}=({a}_{l},{a}_{m},{a}_{u})$ and $\widetilde{B}=\left({b}_{l},{b}_{m},{b}_{u}\right)$ is observed only when ${a}_{l}={b}_{l},{a}_{m}={b}_{m},$ ${a}_{u}={b}_{u}$.

### Definition 3.7^12^

**Fig. 2:**
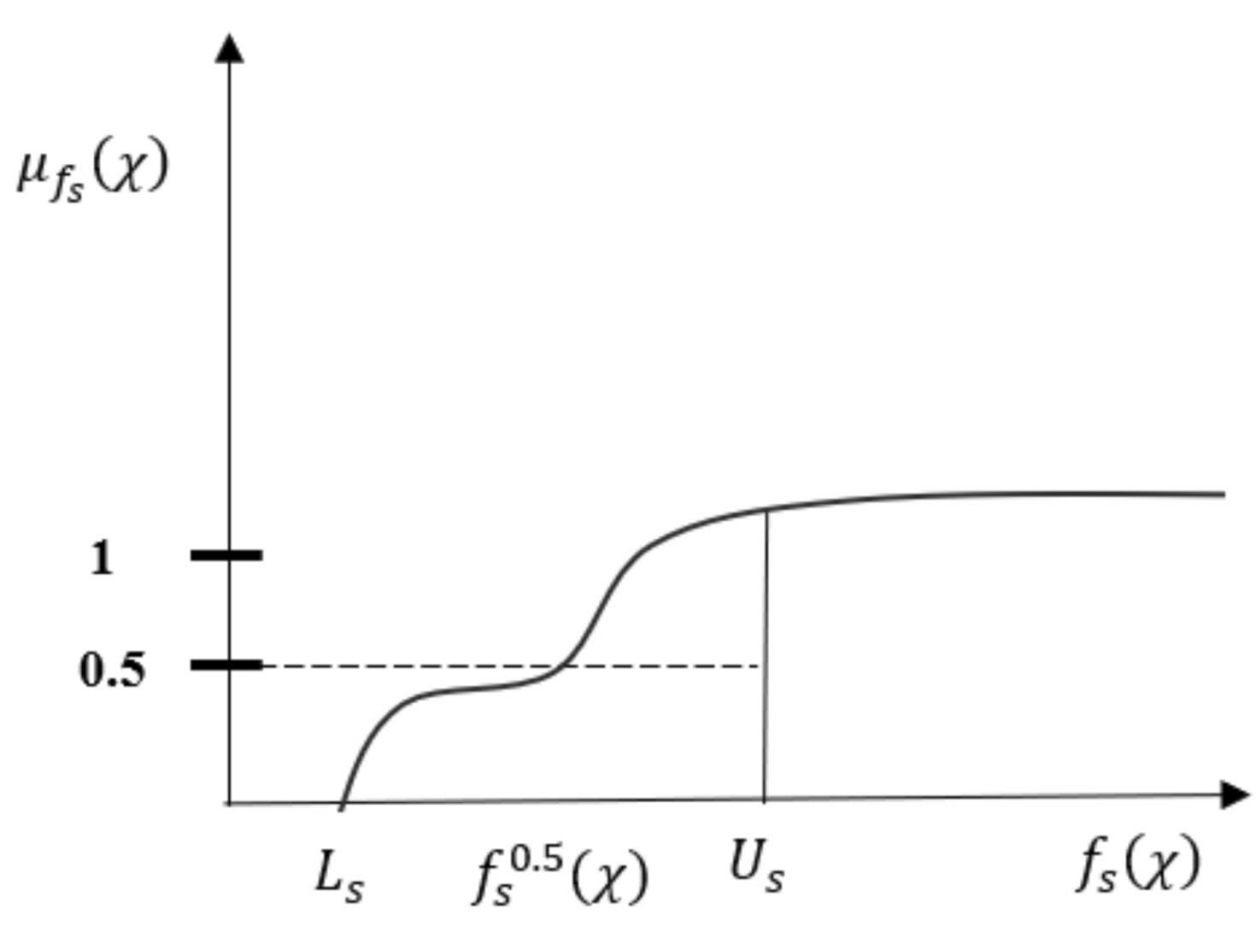
Exponential membership function.

Each objective function is linked to the following exponential membership functions (Fig. 2).

$${\mu }_{{f}_{s}}\left(\chi \right)=\left\{\begin{array}{c}0, for {L}_{s}> {f}_{s}\left(\chi \right)\\ {\alpha }_{s}\left[1-\text{exp}\left\{\frac{(-{\beta }_{s})({U}_{s}-{f}_{s}\left(\chi \right))}{({U}_{s}-{L}_{s})}\right\}\right] for {L}_{s}\le {f}_{s}\left(\chi \right)\le {U}_{s} \\ 1, for {f}_{s}\left(\chi \right)>{U}_{s}\end{array}\right.$$

where ${\alpha }_{s}>1,$ and ${\beta }_{s }>0$. The exponential membership function can be determined by asking the DM to specify the three points ${L}_{s},{f}_{s}^{0.5}(\chi )$ and ${U}_{s}$ such that ${f}_{s}^{min}\left(\chi \right)\le {L}_{s}\le {f}_{s}^{0.5}\left(\chi \right)\le {U}_{s}\le {f}_{s}^{max}\left(\chi \right)$ where ${f}_{s}^{0.5}(\chi )$ represents the value of ${f}_{s}\left(\chi \right)$ such that the degree of membership function ${\mu }_{{f}_{s}}\left(\chi \right)$ is 0.5.

### Definition 3.8

Consider two positive triangular fuzzy numbers, denoted as $\widetilde{A}=({a}_{l},{a}_{m},{a}_{u})$ and $\widetilde{B}=\left({b}_{l},{b}_{m},{b}_{u}\right).$ Hence, fundamental fuzzy arithmetic operations on these fuzzy integers can be established via the subsequent relation

$$\text{Addition}: \widetilde{A}+\widetilde{B}=({a}_{l}+{b}_{l},{a}_{m}+{b}_{m},{a}_{u}+{b}_{u})$$

$$\text{Subtraction}: \widetilde{A}-\widetilde{B}=({a}_{l}-{b}_{l},{a}_{m}-{b}_{m},{a}_{u}-{b}_{u})$$

$$\text{Multiplication}: \widetilde{A}\times \widetilde{B}=({a}_{l}.{b}_{l},{a}_{m}.{b}_{m},{a}_{u}.{b}_{u})$$

$$\text{Division}: \widetilde{A}/\widetilde{B}=({a}_{l}/{b}_{u},{a}_{m}/{b}_{m},{a}_{u}/{b}_{l})$$

### Formulation of the FMOTP model

#### Fuzzy Multi Objective Transportation Problem (FMOTP)

Real-world scenarios give rise to transportation problems known as fuzzy multi-objective transportation problems (FMOTPs). It involves optimizing multiple conflicting objectives while considering certain characteristics as fuzzy integers. Let’s examine a situation where there are $m$ sources with varying quantities of goods that need to be distributed to $n$ distinct destination points. Furthermore, for every route $( \mathfrak{i},\mathfrak{j})$ for all $R$ objectives, the presence of a fuzzy penalty ${\widetilde{C}}_{\mathfrak{i}\mathfrak{j}}^{r} \left({\text{where}} \mathfrak{i}=\text{1,2},\dots ,m;\mathfrak{j}=\text{1,2},\dots ,n;r=\text{1,2},\dots ,R\right)$ is considered. To effectively carry commodities from diverse supply sources to designated demand areas, it is essential to formulate a pragmatic transportation plan that adeptly reconciles several opposing objectives. In Model 1, the transportation of goods from sources $\mathfrak{i}$ to destinations $\mathfrak{j}$ while taking into account multiple objectives $R$ is represented by ${\chi }_{\mathfrak{i}\mathfrak{j}}$ in the Fuzzy Multi-Objective Transportation Problem (FMOTP).

In Model 1, the Fuzzy Multi-Objective Transportation Problem (FMOTP) represents ${\chi }_{\mathfrak{i}\mathfrak{j}}$ as the transportation of products from sources $\mathfrak{i}$ to destinations $\mathfrak{j}$, considering multiple objectives $R$.

#### The FMOTP’s Mathematical Model

##### Model 1

Optimize $${Z}_{r}=\sum_{\mathfrak{i}=1}^{m}\sum_{\mathfrak{j}=1}^{n}{\widetilde{C}}_{\mathfrak{i}\mathfrak{j}}^{r}{\chi }_{\mathfrak{i}\mathfrak{j}} , r=\text{1,2},\dots ,R$$

Subject to

3 $${\sum }_{\mathfrak{j}=1}^{n}{\chi }_{\mathfrak{i}\mathfrak{j}}={a}_{\mathfrak{i}}$$

4 $${\sum }_{\mathfrak{i}=1}^{m}{\chi }_{\mathfrak{i}\mathfrak{j}}={b}_{\mathfrak{j}}$$

$${\chi }_{\mathfrak{i}\mathfrak{j}}\ge 0 , \mathfrak{i}=\text{1,2},\dots ,m \mathfrak{j}=\text{1,2},\dots ,n$$

The variable ${\chi }_{\mathfrak{i}\mathfrak{j}}$ in the FMOTP denotes the quantity of goods being transported from the origin $\mathfrak{i}$ to the destination $\mathfrak{j}$. The variables ${a}_{\mathfrak{i}}$ and ${b}_{\mathfrak{j}}$ denote the quantity supplied at origin $\mathfrak{i}$ and the quantity demanded at destination $\mathfrak{j}$, respectively.

### FLP technique: fuzzy linear programming

One of the key methods for solving multi-objective transportation problems that is available in the literature is fuzzy linear programming. This strategy was first developed by Zimmermann^4^. To better understand this method, let’s examine an example of a crips multi-objective transportation problem (CMOTP) such as,

Optimize

$${Z}_{r}=\sum_{\mathfrak{i}=1}^{m}\sum_{\mathfrak{j}=1}^{n}{C}_{\mathfrak{i}\mathfrak{j}}^{r}{\chi }_{\mathfrak{i}\mathfrak{j}} , r=\text{1,2},\dots ,R$$

Subject to

$${\sum }_{\mathfrak{j}=1}^{n}{\chi }_{\mathfrak{i}\mathfrak{j}}={a}_{\mathfrak{i}}$$

$${\sum }_{\mathfrak{i}=1}^{m}{\chi }_{\mathfrak{i}\mathfrak{j}}={b}_{\mathfrak{j}}$$

$${\chi }_{\mathfrak{i}\mathfrak{j}}\ge 0 , \mathfrak{i}=\text{1,2},\dots ,m \mathfrak{j}=\text{1,2},\dots ,n$$

The fuzzy linear membership function, denoted as ${\mu }_{r}\left({Z}_{r}\left(\chi \right)\right)$ in the FMOTP framework, links the best ${L}_{r}$ and the worst ${U}_{r}$ of each objective function ${Z}_{r}\left(\chi \right)$. This function evaluates the membership degree or satisfaction level of a given solution $\chi$ within the range specified by ${L}_{r}$ and ${U}_{r}$. When ${L}_{r}\ne {U}_{r} ,$ the fuzzy linear membership function in the case of minimization is given by

$${\mu }_{r}\left({Z}_{r}\left(\chi \right)\right)=\left\{\begin{array}{c}1, if {Z}_{r}\left(\chi \right)\le {L}_{r}\\ \frac{{U}_{r}-{Z}_{r}\left(\chi \right)}{{U}_{r}-{L}_{r}}, if {L}_{r}\le {Z}_{r}\left(\chi \right)\le {U}_{r} \\ 0, if {Z}_{r}\left(\chi \right)\ge {U}_{r}\end{array}\right.$$

and in the case of maximization is given by

$${\mu }_{r}\left({Z}_{r}\left(\chi \right)\right)=\left\{\begin{array}{c}0, if {Z}_{r}\left(\chi \right)\le {L}_{r}\\ \frac{{Z}_{r}\left(\chi \right)-{L}_{r}}{{U}_{r}-{L}_{r}}, if {L}_{r}\le {Z}_{r}\left(\chi \right)\le {U}_{r} \\ 1, if {Z}_{r}\left(\chi \right)\ge {U}_{r}\end{array}\right.$$

If $${L}_{r}={U}_{r}$$ then $${\mu }_{r}\left({Z}_{r}\left(\chi \right)\right)=1, \forall r=\text{1,2},\dots ,R.$$

Using the membership function, define a parameter λ as the level of acceptance of a solution.

## Methodology proposal

The proposed methodology operates in two primary phases. Initially, a parametric equation is created using fuzzy parameters to convert the FMOTP into a deterministic MOTP with multiple divisions. The parametric equation incorporates a membership parameter, $\mu$, constrained to the interval $\left[\text{0,1}\right].$ Varying the values of $\mu$ within this range generates distinct versions of multi-objective transportation problems for different divisions, with each value of $\mu$ producing a unique problem configuration.

All the CMOTPs with various splits built using different precision values $\mu$ are solved using FLP techniques in the second phase. Below is a detailed explanation of the stepwise algorithm and the formulation of the model:

### Formulate the model of FMOTP with the assistance of FPP

Triangular fuzzy numbers (TFNs) can be used to describe the parameter ${\widetilde{C}}_{\mathfrak{i}\mathfrak{j}}^{r}$ in Model 1 since it is considered a fuzzy parameter. A fuzzy number is considered normalized if there exist one point $\chi \in X$ such that ${\mu }_{\widetilde{Q}}(\chi )=1$. The entire membership function can be divided up into distinct divides using these points. To gain a better understanding of this idea, assume that the triangular fuzzy numbers ${\widetilde{C}}_{\mathfrak{i}\mathfrak{j}}^{r}$ given in Model 1 are represented by the membership function ${\mu }_{{\widetilde{C}}_{\mathfrak{i}\mathfrak{j}}^{r}}$. Rather than delineating the model formulation for distinct parameters ${\widetilde{C}}_{\mathfrak{i}\mathfrak{j}}^{r}$, the parameters in Model 1 may be represented as $\widetilde{Q}=({q}^{l},{q}^{m},{q}^{u})$, where the exponential membership function is denoted by ${\mu }_{\widetilde{Q}}$ as follows:

$${\mu }_{\widetilde{Q}}=1 (\text{at point of division})\text{ and }{\mu }_{\widetilde{Q}}=0, (\text{otherwise})$$

$${\mu }_{\widetilde{Q}}(q)={a}_{Q}\left[1-\text{exp}\left\{\frac{(-{b}_{Q})(q-{q}^{l})}{({q}^{m}-{q}^{l})}\right\}\right] (\text{for left split})$$

$${\mu }_{\widetilde{Q}}(q)={a}_{Q}\left[1-\text{exp}\left\{\frac{(-{b}_{Q})({q}^{u}-q)}{({q}^{u}-{q}^{m})}\right\}\right] (\text{for right split})$$

where ${b}_{Q}>0$ or ${b}_{Q}<0$ and ${a}_{Q}=1/(1-\text{exp}\left\{-{b}_{Q}\right\})$. Which in terms of $q$ can be written as;

5 $$q={q}^{l}+\left\{\frac{\left({q}^{m}-{q}^{l}\right)}{(-{b}_{Q})}\right\}\text{log}(1-\frac{{\mu }_{\widetilde{Q}}}{{a}_{Q}})$$

6 $$q={q}^{u}-\left\{\frac{\left({q}^{u}-{q}^{m}\right)}{(-{b}_{Q})}\right\}\text{log}(1-\frac{{\mu }_{\widetilde{Q}}}{{a}_{Q}})$$

The membership grade, denoted as ${\mu }_{\widetilde{Q}}$, is regarded as an accuracy parameter in the parametric equations expressed in Eqs. (5) and (6). It is situated inside the closed interval $[\text{0,1}]$. Any values of $\widetilde{Q}$ between ${q}^{l}$ and ${q}^{u}$ are obtained, including them, by varying ${\mu }_{\widetilde{Q}}$ in $[\text{0,1}]$. Using the parametric equation provided in Eqs. (5) and (6), **Model 1** can now be formulated in terms of ${\widetilde{C}}_{\mathfrak{i}\mathfrak{j}}^{r}$ using the steps shown below:

#### Model 2 (For left split)

Optimize

$${Z}_{r}^{\mathcal{L}}=\sum_{\mathfrak{i}=1}^{m}\sum_{\mathfrak{j}=1}^{n}({C}_{\mathfrak{i}\mathfrak{j}}^{rl}+\left\{\frac{\left({C}_{\mathfrak{i}\mathfrak{j}}^{rm}-{C}_{\mathfrak{i}\mathfrak{j}}^{rl}\right)}{(-{b}_{C})}\right\}\text{log}(1-\frac{{\mu }_{\widetilde{C}}}{{a}_{C}})){\chi }_{\mathfrak{i}\mathfrak{j}} , r=\text{1,2},\dots ,R$$

Subject to

$${\sum }_{\mathfrak{j}=1}^{n}{\chi }_{\mathfrak{i}\mathfrak{j}}={a}_{\mathfrak{i}}$$

$${\sum }_{\mathfrak{i}=1}^{m}{\chi }_{\mathfrak{i}\mathfrak{j}}={b}_{\mathfrak{j}}$$

$${\chi }_{\mathfrak{i}\mathfrak{j}}\ge 0, \mathfrak{i}=\text{1,2},\dots ,m \mathfrak{j}=\text{1,2},\dots ,n$$

#### Model 3 (For right split)

Optimize

$${Z}_{r}^{\mathfrak{R}}=\sum_{\mathfrak{i}=1}^{m}\sum_{\mathfrak{j}=1}^{n}({C}_{\mathfrak{i}\mathfrak{j}}^{ru}-\left\{\frac{\left({C}_{\mathfrak{i}\mathfrak{j}}^{ru}-{C}_{\mathfrak{i}\mathfrak{j}}^{rm}\right)}{(-{b}_{C})}\right\}\text{log}(1-\frac{{\mu }_{\widetilde{C}}}{{a}_{C}})){\chi }_{\mathfrak{i}\mathfrak{j}}, r=\text{1,2},\dots ,R$$

Subject to

$${\sum }_{\mathfrak{j}=1}^{n}{\chi }_{\mathfrak{i}\mathfrak{j}}={a}_{\mathfrak{i}}$$

$${\sum }_{\mathfrak{i}=1}^{m}{\chi }_{\mathfrak{i}\mathfrak{j}}={b}_{\mathfrak{j}}$$

$${\chi }_{\mathfrak{i}\mathfrak{j}}\ge 0 , \mathfrak{i}=\text{1,2},\dots ,m \mathfrak{j}=\text{1,2},\dots ,n$$

Considering that only the solutions that achieve every goal are included in a fuzzy decision set. One way to rephrase the Model 3 and Model 4 is as follows.

#### Model 4 (For left split)

Optimize

$${Z}_{r}^{\mathcal{L}}=\sum_{\mathfrak{i}=1}^{m}\sum_{\mathfrak{j}=1}^{n}({C}_{\mathfrak{i}\mathfrak{j}}^{rl}+\left\{\frac{\left({C}_{\mathfrak{i}\mathfrak{j}}^{rm}-{C}_{\mathfrak{i}\mathfrak{j}}^{rl}\right)}{(-b)}\right\}\text{log}(1-\frac{\mu }{a})){\chi }_{\mathfrak{i}\mathfrak{j}}, r=\text{1,2},\dots ,R$$

Subject to

$${\sum }_{\mathfrak{j}=1}^{n}{\chi }_{\mathfrak{i}\mathfrak{j}}={a}_{\mathfrak{i}}$$

$${\sum }_{\mathfrak{i}=1}^{m}{\chi }_{\mathfrak{i}\mathfrak{j}}={b}_{\mathfrak{j}}$$

$${\chi }_{\mathfrak{i}\mathfrak{j}}\ge 0 , \mathfrak{i}=\text{1,2},\dots ,m \mathfrak{j}=\text{1,2},\dots ,n$$

where $${\varvec{a}}=1/(1-\mathbf{exp}\left\{-{\varvec{b}}\right\})$$**,** $${\varvec{b}}=0.8$$.

#### Model 5 (For right split)

Optimize

$${Z}_{r}^{\mathfrak{R}}=\sum_{\mathfrak{i}=1}^{m}\sum_{\mathfrak{j}=1}^{n}({C}_{\mathfrak{i}\mathfrak{j}}^{ru}-\left\{\frac{\left({C}_{\mathfrak{i}\mathfrak{j}}^{ru}-{C}_{\mathfrak{i}\mathfrak{j}}^{rm}\right)}{(-b)}\right\}\text{log}(1-\frac{\mu }{a})){\chi }_{\mathfrak{i}\mathfrak{j}} , r=\text{1,2},\dots ,R$$

Subject to

$${\sum }_{\mathfrak{j}=1}^{n}{\chi }_{\mathfrak{i}\mathfrak{j}}={a}_{\mathfrak{i}}$$

$${\sum }_{\mathfrak{i}=1}^{m}{\chi }_{\mathfrak{i}\mathfrak{j}}={b}_{\mathfrak{j}}$$

$${\chi }_{\mathfrak{i}\mathfrak{j}}\ge 0 , \mathfrak{i}=\text{1,2},\dots ,m \mathfrak{j}=\text{1,2},\dots ,n$$

##### Theorem

*In terms of functionality, Model 1 is comparable to both Model 4 and Model 5.*

##### *Proof*

The equivalence between Model 1 and Models 4 and 5 can be demonstrated by establishing the equivalence between the sets of feasible and optimal solutions. Let $F$ represent the feasible solution set for Model 1, and let ${F}^{*}$ represent the combined feasible solution sets for Models 4 and 5. If the solution for Model 1, $X=\left\{{\widetilde{\chi }}_{\mathfrak{i}\mathfrak{j}}\right\}\in {F}^{*}$, then clearly $X\in F$, and the converse is also true. Therefore, $F={F}^{*}$. Therefore, it follows that Model 1’s feasible set is equivalent to Models 4 and 5.

IT will BE demonstrated that the optimal solution sets for Models 4 and 5 are identical to those of Model 1. Let ${X}^{*}=\left\{{\widetilde{\chi }}_{\mathfrak{i}\mathfrak{j}}^{*}\right\}$ represent the optimal solution for Model 1, where ${X}^{*}\in F$, and X represents the ideal solution of Model 1. If it is established that $F={F}^{*}$, it follows that. On the other hand, if ${X}^{*}$ is not optimal in ${F}^{*}$, there must be some element $X\in {F}^{*}$ such that

$${Z}_{r}^{\mathcal{L}}X\le {Z}_{r}^{\mathcal{L}}{X}^{*}\text{ and }{Z}_{r}^{\mathfrak{R}}X\le {Z}_{r}^{\mathfrak{R}}{X}^{*}$$

and hence

$$\sum_{\mathfrak{i}=1}^{m}\sum_{\mathfrak{j}=1}^{n}({C}_{\mathfrak{i}\mathfrak{j}}^{rl}+\left\{\frac{\left({C}_{\mathfrak{i}\mathfrak{j}}^{rm}-{C}_{\mathfrak{i}\mathfrak{j}}^{rl}\right)}{(-b)}\right\}\text{log}\left(1-\frac{\mu }{a}\right))X\le \sum_{\mathfrak{i}=1}^{m}\sum_{\mathfrak{j}=1}^{n}({C}_{\mathfrak{i}\mathfrak{j}}^{rl}+\left\{\frac{\left({C}_{\mathfrak{i}\mathfrak{j}}^{rm}-{C}_{\mathfrak{i}\mathfrak{j}}^{rl}\right)}{(-b)}\right\}\text{log}\left(1-\frac{\mu }{a}\right)) {X}^{*}$$

and

$$\sum_{\mathfrak{i}=1}^{m}\sum_{\mathfrak{j}=1}^{n}({C}_{\mathfrak{i}\mathfrak{j}}^{ru}-\left\{\frac{\left({C}_{\mathfrak{i}\mathfrak{j}}^{ru}-{C}_{\mathfrak{i}\mathfrak{j}}^{rm}\right)}{(-b)}\right\}\text{log}(1-\frac{\mu }{a})X\le \sum_{\mathfrak{i}=1}^{m}\sum_{\mathfrak{j}=1}^{n}({C}_{\mathfrak{i}\mathfrak{j}}^{ru}-\left\{\frac{\left({C}_{\mathfrak{i}\mathfrak{j}}^{ru}-{C}_{\mathfrak{i}\mathfrak{j}}^{rm}\right)}{(-b)}\right\}\text{log}(1-\frac{\mu }{a}) {X}^{*}$$

This leads to a contradiction because, in $F$, ${X}^{*}$ is the best solution even if the formulas $\left({C}_{\mathfrak{i}\mathfrak{j}}^{rl}+\left\{\frac{\left({C}_{\mathfrak{i}\mathfrak{j}}^{rm}-{C}_{\mathfrak{i}\mathfrak{j}}^{rl}\right)}{(-b)}\right\}\text{log}\left(1-\frac{\mu }{a}\right)\right)and ({C}_{\mathfrak{i}\mathfrak{j}}^{ru}-\left\{\frac{\left({C}_{\mathfrak{i}\mathfrak{j}}^{ru}-{C}_{\mathfrak{i}\mathfrak{j}}^{rm}\right)}{(-b)}\right\}\text{log}\left(1-\frac{\mu }{a}\right))$ can take on any real number between ${C}_{\mathfrak{i}\mathfrak{j}}^{rl}$ to ${C}_{\mathfrak{i}\mathfrak{j}}^{rm}$ and ${C}_{\mathfrak{i}\mathfrak{j}}^{rm}$ to ${C}_{\mathfrak{i}\mathfrak{j}}^{ru}$, respectively. Then, since ${X}^{*}$ represents the optimal solution for Model 1, the following inequality holds

$$\sum_{\mathfrak{i}=1}^{m}\sum_{\mathfrak{j}=1}^{n}({C}_{\mathfrak{i}\mathfrak{j}}^{rl},{C}_{\mathfrak{i}\mathfrak{j}}^{rm},{C}_{\mathfrak{i}\mathfrak{j}}^{ru}){X}^{*}\le \sum_{\mathfrak{i}=1}^{m}\sum_{\mathfrak{j}=1}^{n}({C}_{\mathfrak{i}\mathfrak{j}}^{rl},{C}_{\mathfrak{i}\mathfrak{j}}^{rm},{C}_{\mathfrak{i}\mathfrak{j}}^{ru})X$$

for any real value between ${C}_{\mathfrak{i}\mathfrak{j}}^{rl}$ to ${C}_{\mathfrak{i}\mathfrak{j}}^{ru}$. Consequently, it is erroneous to presume that ${X}^{*}$ is not the best solution in ${F}^{*}$. This conclusion serves as a valid proof of the theorem.

### The proposed algorithm

**Fig. 3:**
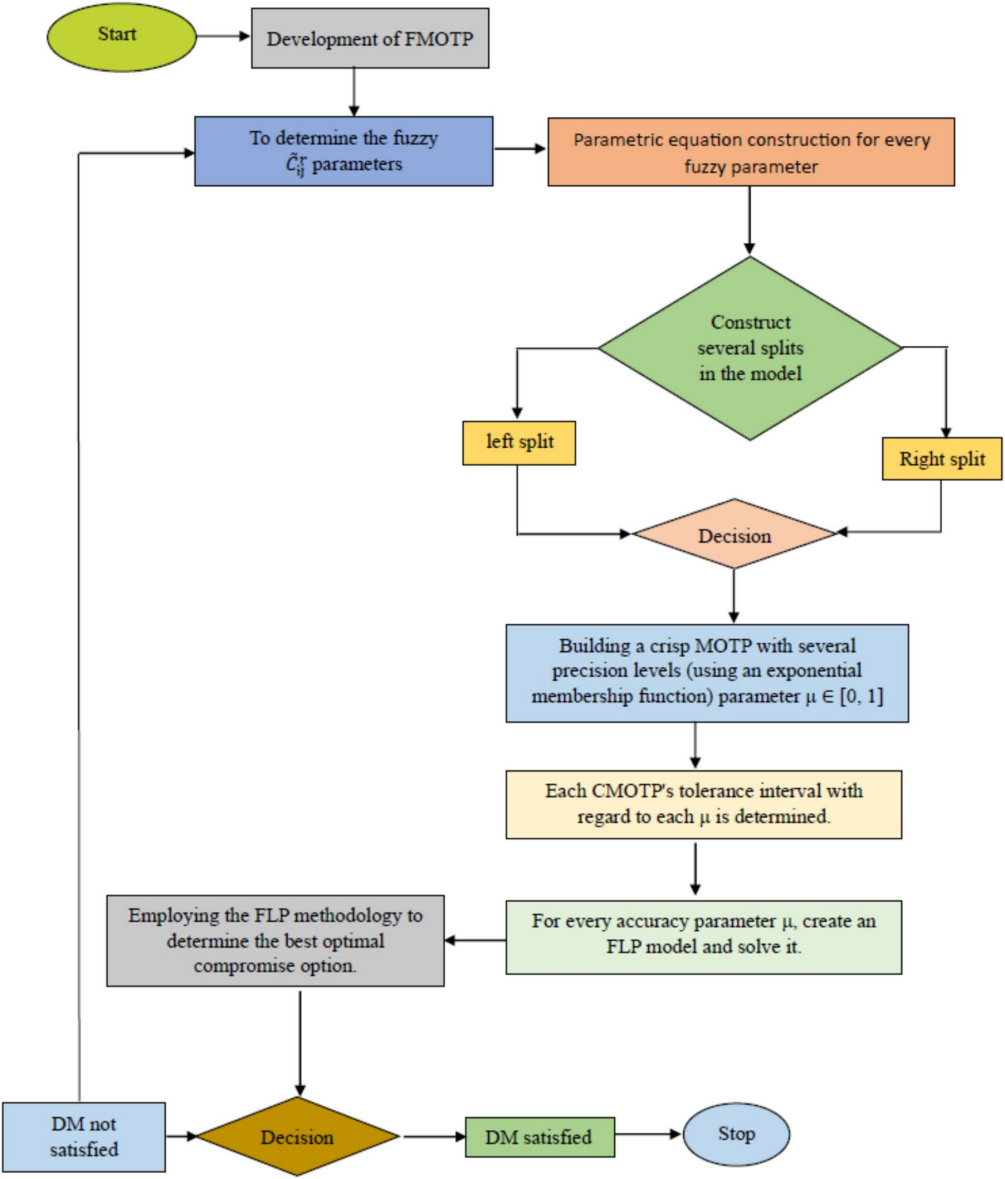
Flowchart for the proposed approach.

Step 1 Determine which parameters are associated with model 1 and use the accuracy parameter $\mu \in \left[\text{0,1}\right]$ to build the parametric equation linked to these parameters.
Step 2 Apply multiple divisions to Model 1 by using the parametric equation that was generated in Step 1. That is, the left split corresponds to Model 4, and the right split corresponds to Model 5.
Step 3 By changing the parameter $\mu \in [\text{0,1}]$ for every division, create several crisp multi-objective transportation problems (CMOTP).
Step 4 Obtain various optimal solutions for different splits by solving the multiple CMOTP generated in Step 3 (${\varvec{b}}=0.8).$
Step 5 From the several compromise solutions produced in Step 4 for each division, choose the best one using the Fuzzy Linear Programming (FLP) technique.
Step 6 The preferred compromise optimal solution and the ideal solution associated with each division split should be measured and their Euclidean distances converted. The optimal compromise solution is found by choosing the answer that yields the lowest Euclidean distance in every split. The proposed algorithm is shown in Fig. 3.

### Advantages of the proposed algorithm

The proposed fuzzy optimization framework offers several key advantages in addressing transportation and logistics challenges:

- Fuzzy models better properly represent operational reality by embracing the dynamic and sometimes non-linear characteristics of logistics environments, in contrast to classic deterministic methods.
- The framework facilitates the concurrent optimization of conflicting objectives—namely cost reduction, time efficiency, and service level enhancement—promoting more thorough and equitable decision-making.
- The model adeptly incorporates imprecise, ambiguous, or partial data through fuzzy sets and membership functions, which are prevalent in real-world logistics contexts.
- This approach generates a collection of compromise solutions at differing confidence levels, offering decision-makers a spectrum of viable options aligned with diverse risk tolerances and operational priorities.

Together, these features enhance the robustness, adaptability, and practical applicability of the proposed fuzzy multi-objective transportation framework.

### Managerial insights

This study provides numerous practical insights and consequences for decision-makers and practitioners in sectors where logistical efficiency, cost management, and uncertainty mitigation are essential.

- The proposed methodology empowers logistics managers to make informed decisions despite imprecise or variable cost, supply, and demand data. This is especially beneficial in volatile markets or crisis-response logistics, where decisions must be taken with incomplete information.
- By adjusting the accuracy parameter μ, managers can model situations with differing degrees of confidence in the input data. This facilitates comprehensive planning by pinpointing solutions that maintain stability even in worst-case scenarios, thus improving operational resilience.
- The concept offers compromise solutions among several objectives (money, time, service), enabling organizations to optimize trade-offs rather than concentrating on a singular aim. This results in more equitable and economical logistical techniques in implementation.
- For organizations managing several suppliers, unpredictable lead times, or inconsistent demand projections, the application of Triangular Fuzzy Numbers (TFNs) facilitates more accurate modeling without depending on inaccurate precise inputs.
- The methodology, executed in LINGO and transferable to other optimization platforms, provides a scalable computational instrument that organizations may integrate into their decision-support systems for enduring strategic logistics optimization.
- The comparative benchmarking of classic and sophisticated methods (e.g., fuzzy DEA, GRA) enables organizations to validate the use of fuzzy approaches by illustrating enhanced proximity to optimal outcomes and increased ranking resilience.

## Numerical example

This study demonstrates the proposed algorithms through the use of a numerical example. This numerical illustration consists of a total of eight origins and three endpoints. The data pertaining to transportation costs, shipments’ value, and profits are expressed as triangular fuzzy sets, symbolised as $\widetilde{Q}=\left({q}^{l},{q}^{m},{q}^{u}\right),$ and may be located in Table 3. The precise quantities of Supplies ${a}_{\mathfrak{i}} (\mathfrak{i}=\text{1,2},\dots ,m)$ and Demands ${b}_{\mathfrak{j}} (\mathfrak{j}=\text{1,2},\dots ,n)$ are also specified in Table 3 (Tables 4, 5).

**Table 3:** Data for the given numerical example.

	${b}_{1}=30$	${b}_{2}=25$	${b}_{3}=14$	
Cost of shipment	$(\text{525,531,540})$	$(\text{425,431,436})$	$(\text{390,395,410})$	${a}_{1}=10$
Value of consignment	$(\text{3400,3500,3550})$	$(\text{372,380,390})$	$(\text{3800,3950,4050})$	
Operating profit	$(\text{480,500,510})$	$(\text{590,600,615})$	$(\text{380,400,410})$	
Cost of shipment	$(\text{386,394,411})$	$(\text{410,418,425})$	$(\text{505,512,520})$	${a}_{2}=13$
Value of consignment	$(\text{2800,2850,2940})$	$(\text{2380,2395,2410})$	$(\text{2500,2590,2620})$	
Operating profit	$(\text{590,600,615})$	$(\text{685,700,710})$	$(\text{480,485,500})$	
Cost of shipment	$(\text{400,405,412})$	$(\text{505,512,520})$	$(\text{400,412,420})$	${a}_{3}=11$
Value of consignment	$(\text{305,310,320})$	$(\text{400,409,415})$	$(\text{380,390,405})$	
Operating profit	$(\text{790,800,815})$	$(\text{970,1000,1050})$	$(\text{1000,1100,1150})$	
Cost of shipment	$(\text{350,355,365})$	$(\text{490,493,500})$	$(\text{560,570,590})$	${a}_{4}=7$
Value of consignment	$(\text{275,290,295})$	$(\text{370,385,400})$	$(\text{412,419,425})$	
Operating profit	$(\text{700,705,715})$	$(\text{600,617,630})$	$(\text{500,518,525})$	
Cost of shipment	$(\text{294,299,304})$	$(\text{380,398,412})$	$(\text{300,315,320})$	${a}_{5}=9$
Value of consignment	$(\text{410,415,425})$	$(\text{500,512,520})$	$(\text{250,255,270})$	
Operating profit	$(\text{580,585,595})$	$(\text{475,490,500})$	$(\text{365,380,390})$	
Cost of shipment	$(\text{314,319,324})$	$(\text{458,464,472})$	$(\text{430,435,450})$	${a}_{6}=9$
Value of consignment	$(\text{508,512,520})$	$(\text{202,215,220})$	$(\text{345,355,360})$	
Operating profit	$(\text{480,488,496})$	$(\text{300,305,320})$	$(\text{505,512,520})$	
Cost of shipment	$(\text{614,619,625})$	$(\text{480,490,510})$	$(\text{350,354,365})$	${a}_{7}=4$
Value of consignment	$(\text{606,612,620})$	$(\text{500,510,520})$	$(\text{500,550,580})$	
Operating profit	$(\text{614,619,625})$	$(\text{490,505,512})$	$(\text{480,490,510})$	
Cost of shipment	$(\text{450,456,462})$	$(\text{382,394,402})$	$(\text{430,439,445})$	${a}_{8}=6$
Value of consignment	$(\text{290,299,305})$	$(\text{500,512,520})$	$(\text{490,499,510})$	
Operating profit	$(\text{595,601,605})$	$(\text{424,432,440})$	$(\text{505,519,530})$

**Table 4:** Sensitivity analysis of problem outcomes in both left and right split situations for various accuracy parameters μ (proposed method).

Code iteration	Split level	Accuracy parameter (${\varvec{\mu}})$	Compromise optimal solution	Corresponding optimal solution	Distance b/w compromise optimal solution and corresponding optimal solution	Preferred solution (proposed method)
1	LS	$0.997$	$(\text{27119.33,81968.72,45443.77})$	$(\text{25921.84,98222.28,47982.31})$	$16494.13$	$(\text{27119.33,81968.72,45443.77})$
	RS	$0.81311$	$(\text{27342.53,82157.63,45578.78})$	$(\text{26057.02,98882.02,48080.15})$	$16959.16$	
2	LS	$0.997$	$(\text{27119.33,81968.72,45443.77})$	$(\text{25921.84,98222.28,47982.31})$	$16494.13$	$(\text{27119.33,81968.72,45443.77})$
	RS	$0.83315$	$(\text{27326.68,82118.12,45551.43})$	$(\text{26044,98818.01,48052.14})$	$16934.73$	
3	LS	$0.99685$	$(\text{27119.24,81967.93,45443.42})$	$(\text{25921.74,98221.69,47981.98})$	$16494.33$	$(\text{27119.24,81967.93,45443.42})$
	RS	$0.83187$	$(\text{27327.7,82120.67,45553.19})$	$(\text{26044.84,98822.14,48053.95})$	$16936.31$	
4	LS	$0.98697$	$(\text{27113.31,81916.71,45420.45})$	$(\text{25914.69,98183.40,47960.13})$	$16507.33$	$(\text{27113.31,81916.71,45420.45})$
	RS	$0.83222$	$(\text{27327.43,82119.96,45552.71})$	$(\text{26044.61,98821.01,48053.46})$	$16935.89$	
5	LS	$0.96156$	$(\text{27098.38,81787.73,45362.63})$	$(\text{25896.96,98086.96,47905.10})$	$16540.03$	$(\text{27098.38,81787.73,45362.63})$
	RS	$0.81871$	$(\text{27338.05,82144.19,45571.37})$	$(\text{26053.41,98864.26,48072.38})$	$16954.83$	
6	LS	$0.98844$	$(\text{27114.18,81924.31,45423.85})$	$(\text{25915.74,98189.07,47963.36})$	$16505.39$	$(\text{27114.18,81924.31,45423.85})$
	RS	$0.82998$	$(\text{27328.66,82131.60,45554.97})$	$(\text{26046.08,98828.22,48053.61})$	$16931.19$	
7	LS	$0.99548$	$(\text{27118.41,81960.80,45440.22})$	$(\text{25920.75,98216.36,47978.93})$	$16496.14$	$(\text{27118.41,81960.80,45440.22})$
	RS	$0.82773$	$(\text{27331,82128.91,45558.88})$	$(\text{26047.55,98835.45,48059.77})$	$16941.37$	
8	LS	$0.99948$	$(\text{27120.83,81981.69,45449.58})$	$(\text{25923.63,98231.97,47987.84})$	$16490.84$	$(27120.83,81981.69,45449.58)$
	RS	$0.81398$	$(\text{27341.76,82153.44,45577.79})$	$(\text{26056.46,98879.27,48078.95})$	$16960.58$	
9	LS	$0.9917$	$(\text{27116.13,81941.14,45431.41})$	$(\text{25918.05,98201.68,47970.56})$	$16501.15$	$(\text{27116.13,81941.14,45431.41})$
	RS	$0.83233$	$(\text{27327.34,82119.75,45552.56})$	$(\text{26044.54,98820.65,48053.30})$	$16935.74$	
10	LS	$0.9993$	$(\text{27120.72,81980.73,45449.16})$	$(\text{25923.5,98231.26,47987.44})$	$16491.09$	$(\text{27120.72,81980.73,45449.16})$
	RS	$0.82991$	$(\text{27329.27,82124.56,45555.89})$	$(\text{26046.12,98828.45,48056.71})$	$16938.73$	

Significant values are in bold.

**Table 5:** Sensitivity analysis of problem outcomes in both left and right split situations for various accuracy parameters μ (Nomani’s method).

Code iteration	Split level	Accuracy parameter (${\varvec{\mu}})$	Compromise optimal solution	Corresponding optimal solution	Distance b/w compromise optimal solution and Corresponding optimal solution	Preferred solution (Nomani’s Method)
1	LS	$0.997$	$(\text{25921.84,68797.39,44233.38})$	$(\text{25921.84,98222.28,47982.31})$	$29662.75$	$(25921.84,68797.39,44233.38)$
	RS	$0.81311$	$(\text{26057.02,69089.12,44300.50})$	$(\text{26057.02,98882.02,48080.15})$	$30031.69$	
2	LS	$0.997$	$(\text{25921.84,68797.39,44233.38})$	$(\text{25921.84,98222.28,47982.31})$	$29662.75$	$(\text{25921.84,68797.39,44233.38})$
	RS	$0.83315$	$(\text{26044,69061.21,44275.40})$	$(\text{26044,98818.01,48052.14})$	$29995.51$	
3	LS	$0.99685$	$(\text{25921.74,68796.48,44233.17})$	$(\text{25921.74,98221.69,47981.98})$	$29663.05$	$(\text{25921.74,68796.48,44233.17})$
	RS	$0.83187$	$(\text{26044.84,69063.01,44277.02})$	$(\text{26044.84,98822.14,48053.95})$	$29997.85$	
4	LS	$0.98697$	$(\text{25914.69,68775.48,44214.74})$	$(\text{25914.69,98183.40,47960.13})$	$29645.47$	$(\text{25914.69,68775.48,44214.74})$
	RS	$0.83222$	$(\text{26044.61,69062.52,44276.58})$	$(\text{26044.61,98821.01,48053.46})$	$29997.21$	
5	LS	$0.96156$	$(\text{25896.96,68720.35,44168.61})$	$(\text{25896.96,98086.96,47905.10})$	$29603.38$	$(\text{25896.96,68720.35,44168.61})$
	RS	$0.81871$	$(\text{26053.41,69081.29,44293.55})$	$(\text{26053.41,98864.26,48072.38})$	$30021.74$	
6	LS	$0.98844$	$(\text{25915.74,68777.90,44217.56})$	$(\text{25915.74,98189.07,47963.36})$	$29648.74$	$(\text{25915.74,68777.90,44217.56})$
	RS	$0.82998$	$(\text{26046.08,69065.47,44279.43})$	$(\text{26046.08,98828.22,48053.61})$	$30001.1$	
7	LS	$0.99548$	$(\text{25920.75,68794.06,44230.54})$	$(\text{25920.75,98216.36,47978.93})$	$29660.11$	$(\text{25920.75,68794.06,44230.54})$
	RS	$0.82773$	$(\text{26047.55,69068.66,44282.26})$	$(\text{26047.55,98835.45,48059.77})$	$30005.52$	
8	LS	$0.99948$	$(\text{25923.63,68802.17,44238.11})$	$(\text{25923.63,98231.97,47987.84})$	$29667.72$	$(\text{25923.63,68802.17,44238.11})$
	RS	$0.81398$	$(\text{26056.46,69087.92,44299.42})$	$(\text{26056.46,98879.27,48078.95})$	$30030.14$	
9	LS	$0.9917$	$(\text{25918.05,68785.78,44223.50})$	$(\text{25918.05,98201.68,47970.56})$	$29653.59$	$(\text{25918.05,68785.78,44223.50})$
	RS	$0.83233$	$(\text{26044.54,69062.31,44276.44})$	$(\text{26044.54,98820.65,48053.30})$	$29997.06$	
10	LS	$0.9993$	$(\text{25923.50,68801.85,44237.76})$	$(\text{25923.5,98231.26,47987.44})$	$29667.33$	$(\text{25923.50,68801.85,44237.76})$
	RS	$0.82991$	$(\text{26046.12,69065.76,44279.49})$	$(\text{26046.12,98828.45,48056.71})$	$30001.42$	

Significant values are in bold.

A complete sensitivity analysis was performed to assess the stability and robustness of the proposed two-step parametric technique by altering the accuracy parameter μ within the range [0, 1]. Table 4 encapsulates the results of this research, emphasizing both left split (LS) and right split (RS) criteria for each iteration.

In this context, the accuracy parameter μ regulates the conversion of fuzzy parameters into their precise equivalents. As μ rises, the modified model adopts a more cautious stance, indicating elevated confidence in the foundational fuzzy data. For each μ-level, the fuzzy compromise solution (FCS) is calculated and subsequently compared to the fuzzy ideal solution (FIS) by determining the Euclidean distance between the two. A reduced distance signifies that the acquired solution is nearer to the optimal.

The results unequivocally demonstrate a similar pattern throughout all iterations: the left split consistently produces a lower Euclidean distance than the corresponding right split, signifying that solutions obtained under LS circumstances are systematically nearer to the ideal. In Iteration 8, the distance for LS at μ = 0.99948 is 16,490.84, however the RS at μ = 0.81398 results in a greater distance of 16,960.58. This pattern is consistent over all 10 rounds, validating the superiority and coherence of the left split technique within the suggested method.

Moreover, the compromise solutions generated by the suggested technique were consistently congruent with those deemed preferable according to Nomani’s method in LS instances, highlighting the method’s reliability and efficacy in decision-making. These findings strengthen the reliability of the suggested methodology, confirming its practical efficacy in obtaining near-optimal and stable solutions amidst varied levels of fuzziness. This sensitivity analysis underscores the model’s adaptability to varying degrees of uncertainty and its ability to continuously direct decision-makers toward robust solutions that fit with optimal aims.

Table 6 presents a comparison that shows the objective values of the Nomani’s approach vary slightly depending on the accuracy parameter used for left split. On the other hand, the suggested approach regularly produces compromise optimal solutions for left split that are nearer the ideal value for several Precision parameters. Therefore, in comparison to Nomani’s method, it can be argued that the suggested technique exhibits higher coherence and generates solutions that are closer to the ideal model.

**Table 6:** The comparison of solutions using the TOPSIS approach.

Accuracy parameter (${\varvec{\mu}})$	Corresponding optimal solution	Preferred solution (Proposed Method)	Preferred solution (Nomani’s Method)	Ranking	
				Proposed Method	Nomani’s Method
$0.997$	$(\text{25921.84,98222.28,47982.31})$	$(\text{27119.33,81968.72,45443.77})$	$(\text{25921.84,68797.39,44233.38})$	$0.63915$	$0.35465$
$0.997$	$(\text{25921.84,98222.28,47982.31})$	$(\text{27119.33,81968.72,45443.77})$	$(\text{25921.84,68797.39,44233.38})$	$0.63915$	$0.35465$
$0.99685$	$(\text{25921.74,98221.69,47981.98})$	$(\text{27119.24,81967.93,45443.42})$	$(\text{25921.74,68796.48,44233.17})$	$0.63915$	$0.35464$
$0.98697$	$(\text{25914.69,98183.40,47960.13})$	$(\text{27119.24,81967.93,45443.42})$	$(\text{25914.69,68775.48,44214.74})$	$0.63864$	$0.35463$
$0.96156$	$(\text{25896.96,98086.96,47905.10})$	$(\text{27113.31,81916.71,45420.45})$	$(\text{25896.96,68720.35,44168.61})$	$0.63738$	$0.35467$
$0.98844$	$(\text{25915.74,98189.07,47963.36})$	$(\text{27114.18,81924.31,45423.85})$	$(\text{25915.74,68777.90,44217.56})$	$0.66028$	$0.35462$
$0.99548$	$(\text{25920.75,98216.36,47978.93})$	$(\text{27118.41,81960.80,45440.22})$	$(\text{25920.75,68794.06,44230.54})$	$0.63908$	$0.35465$
$0.99948$	$(\text{25923.63,98231.97,47987.84})$	$(\text{27120.83,81981.69,45449.58})$	$(\text{25923.63,68802.17,44238.11})$	$0.63928$	$0.35464$
$0.9917$	$(\text{25918.05,98201.68,47970.56})$	$(\text{27116.13,81941.14,45431.41})$	$(\text{25918.05,68785.78,44223.50})$	$0.63888$	$0.35464$
$0.9993$	$(\text{27120.72,81980.73,45449.16})$	$(\text{27120.72,81980.73,45449.16})$	$(\text{25923.50,68801.85,44237.76})$	$0.63927$	$0.35464$

## Results and discussion

The following section presents a comprehensive examination of the outcomes achieved by the proposed approach for the aforementioned Example, juxtaposed with the outcomes of other well-established approaches^13,23,24^ . To enhance comprehension, the fuzzy compromise solution that was achieved is transformed into a precise form by utilizing the exponential membership function, which is developed for different fuzzy parameters. The parameter μ is determined using an unpredictable approach in the proposed investigation. The FLP approach—a well acknowledged methodology in the existing literature is used to choose a single FCS from the several options as offered.

This method is constantly applied to all levels of division inherent to the scenario. Each instance of split-level operation results in a distinct fuzzy compromise solution (FCS). Moreover, the process entails identifying a corresponding fuzzy ideal solution (FIS) for every accuracy parameter $\mu$. Identifying the optimal fuzzy compromise solution necessitates calculating the Euclidean distance between the selected fuzzy compromise solution (FCS) and its associated fuzzy ideal solution (FIS). It is the division with the least Euclidean distance to its corresponding FIS that is the best solution. This approach facilitates the identification of a superior FCS, resulting in enhanced resilience and effectiveness in the proposed methodology. This paper presents a sensitivity analysis of the results obtained for Examples in Table 4, when the precision parameter μ is adjusted.

According to the sensitivity analysis table, for all precision values of $\mu$, the Euclidean distance between the fuzzy optimal compromise solution and its associated fuzzy ideal solution continuously falls at the left-split level as opposed to the right-split level. This indicates that the best and most equitable solution is located within the left split of the fuzzy data. Consequently, using the proposed approach and Nomani’s method (Table 5), the optimal compromise solution are $(\text{27120.83,81981.69,45449.58})$ and $(\text{25921.84,68797.39,44233.38})$ achieved for the left split section for the precision value $\mu =0.99948,0.997$ respectively.

The problem was also addressed using the fuzzy DEA strategy as outlined by Bagheri et al.^23,24^, resulting in the solution $(\text{27725.1667,89463.83,44164.50})$. Additionally, the Gery Relationship Analysis (GRA) technique employed by Kacher and Singh yielded the solution $\left(\text{27153,81568,45071}\right)$. Comparing these results, the proposed approach produced solutions of $(\text{27120.83,81981.69,45449.58})$, while the GRA technique resulted in $\left(\text{27153,81568,45071}\right)$, and the fuzzy DEA approach provided $\left(\text{27725.1667,89463.83,44164.50}\right)$ for all three objectives.

The first and third objectives are accomplished more effectively with the proposed method than with the fuzzy DEA approach. Furthermore, the proposed strategy is superior to the linear membership function. Therefore, it fails to improve the value of the second objective because of the inherent compromise among the several goals.

**Fig. 4:**
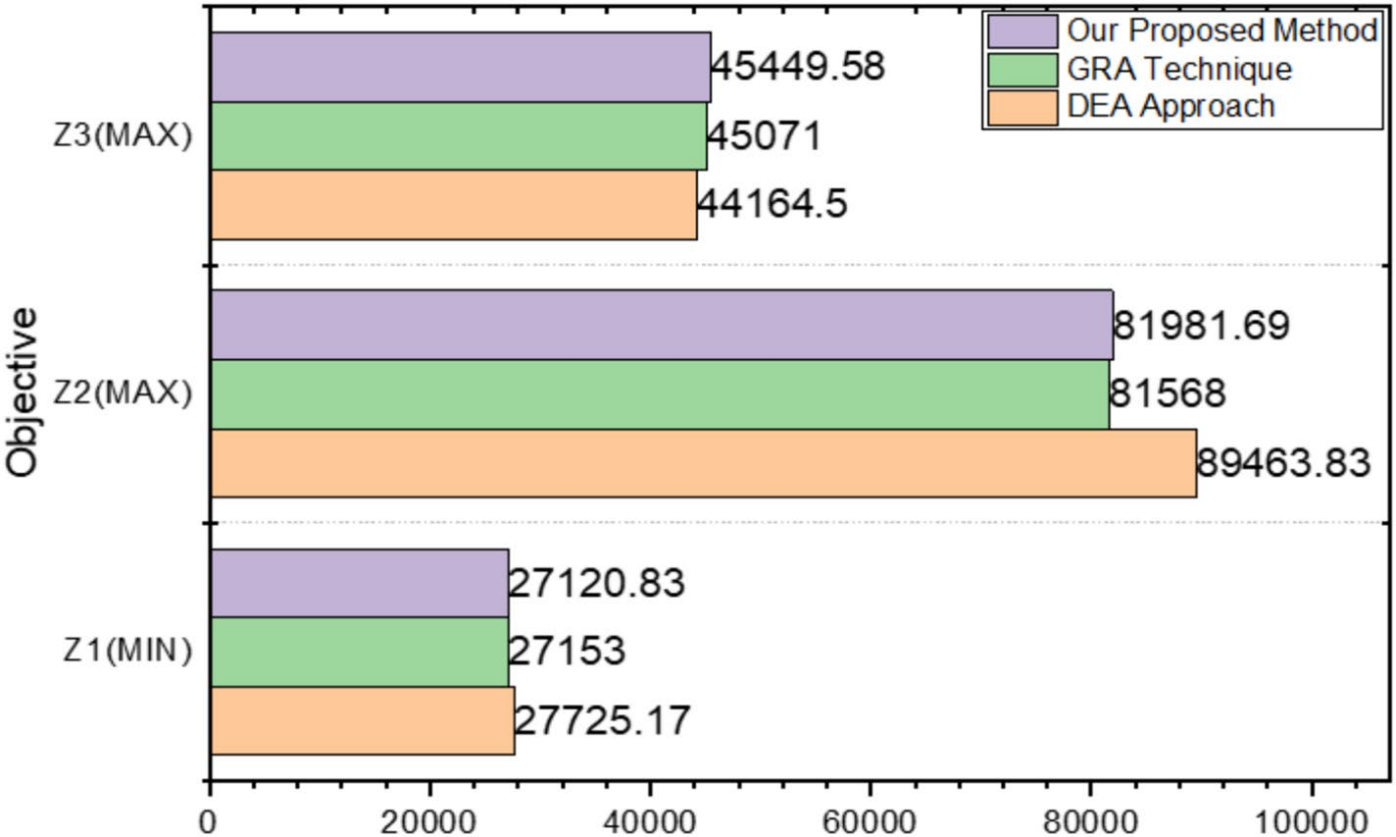
Comparison of acquired results.

**Fig. 5:**
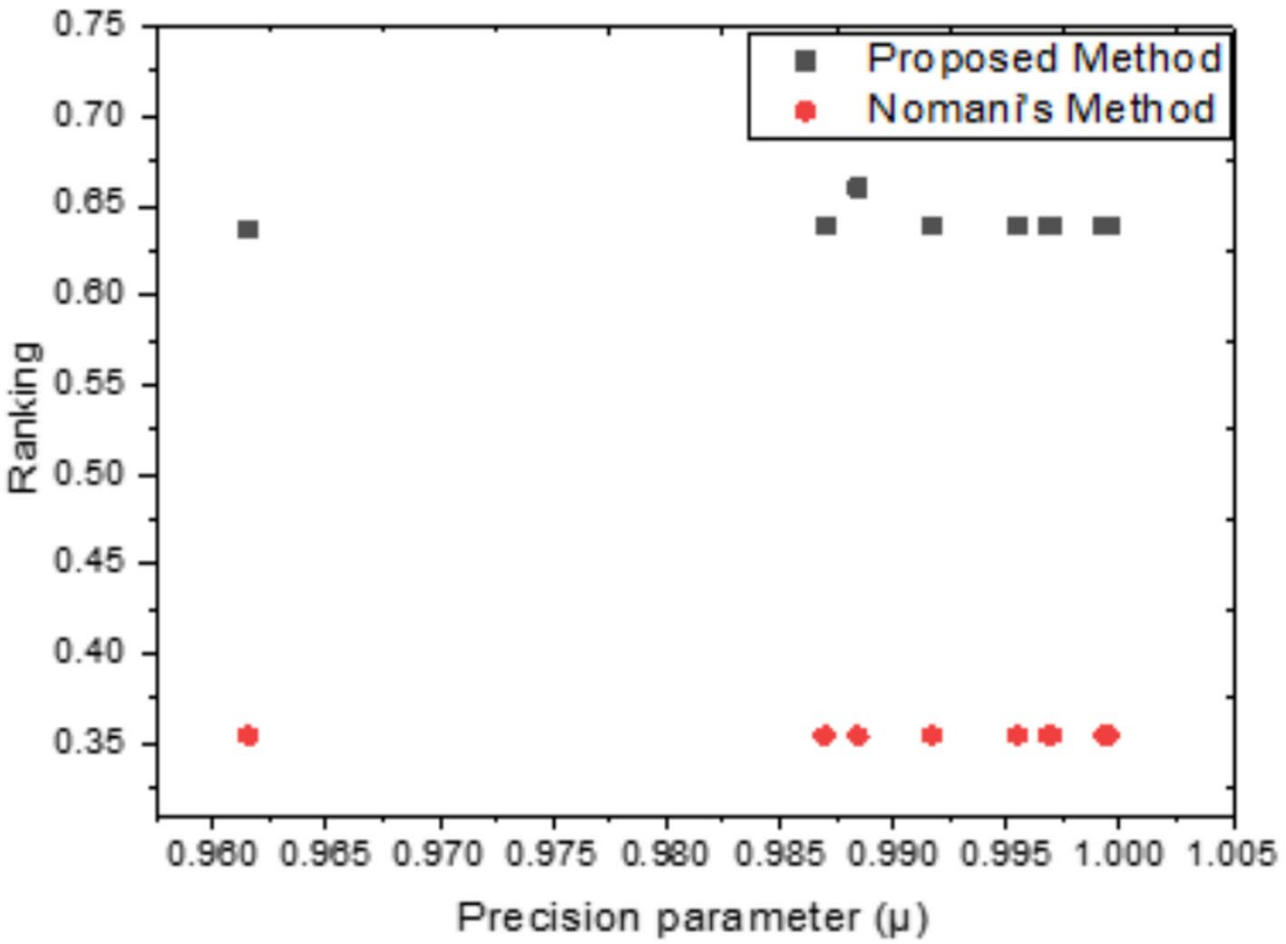
Comparison of the consistency between Nomani’s method and the Proposed method.

The suggested technique has a better achievement outcome, as seen in Fig. 4 that compares the three objective functions ${Z}_{1}$, ${Z}_{2}$ and ${Z}_{3}$ solutions in the two methods (GRA Technique and DEA Approach). Figure 5 that shows how the proposed strategy and Nomani’s method compare in terms of ranking. It is clear that the proposed approach is consistently better than Nomani’s strategy.

## Conclusion

This paper presents a rigorous and practically applicable methodology for addressing Fuzzy Multi-Objective Transportation Problems (FMOTPs), frequently encountered in logistics and supply chain operations amid uncertainty. The suggested two-step parametric method—based on Triangular Fuzzy Numbers (TFNs) and the accuracy parameter μ ∈ [0, 1]—facilitates the conversion of ambiguous issue parameters into Crisp Multi-Objective Transportation Problems (CMOTPs). The solutions are subsequently determined using fuzzy linear programming methods, and the optimal compromise solution is chosen based on its Euclidean distance from the fuzzy ideal. The model, executed via the LINGO 18.0 platform and corroborated with numerical examples derived from previous research^23,24^, exhibits enhanced efficacy relative to established methodologies like Nomani’s method, particularly regarding stability, closeness to optimal solutions, and ranking consistency as assessed by the TOPSIS evaluation.

The practical implications of this study are substantial for industry stakeholders. The methodology facilitates sound decision-making in contexts characterized by inadequate or ambiguous information, enabling supply chain managers to simulate diverse scenarios, evaluate trade-offs among cost, time, and service quality, and improve resilience to disruptions. The model offers several feasible alternatives, allowing for adaptable modifications in strategic and operational plans. Moreover, its organized framework can be included into enterprise resource planning (ERP) systems for real-time logistics enhancement.

Managerial insights encompass enhanced capacity for risk-sensitive planning, decision simulation under uncertainty utilizing the μ parameter, and justification of decisions through clear, data-driven metrics. This improves both efficiency and stakeholder confidence in strategic transportation decisions.

The study has limitations despite its strengths. The model’s dependence on TFNs and an exponential membership function may inadequately represent complex or asymmetric uncertainty distributions. The methodology was confirmed by a singular numerical case study, hence constraining the generalizability of the findings. The LINGO-based approach, although effective, may not be easily available to all practitioners due to license and integration limitations.

Future research ought to investigate alternative membership functions (e.g., quadratic or hyperbolic) to more effectively model intricate uncertainties, implement the framework across diverse industry datasets for comprehensive validation, and contemplate incorporating the method into open-source platforms like Python or MATLAB to improve accessibility and adaptability. Utilizing modern multi-criteria decision-making (MCDM) technologies like VIKOR or PROMETHEE could enhance the assessment and ranking of compromise alternatives.

## Data Availability

All data generated or analysed during this study are included in this published article.
